# Integrated cardiovascular risk management programme versus usual care in patients at high cardiovascular risk: an observational study in general practice

**DOI:** 10.3399/BJGPO.2020.0099

**Published:** 2021-03-16

**Authors:** Suzanne Marchal, Arnoud WJ van 't Hof, Henk JG Bilo, Sander J Deijns, Jan Evert Heeg, Marieke Schoenmakers, Michiel Schouwink, Olof Schwantje, Michiel L Bots, Arno W Hoes, Monika Hollander

**Affiliations:** 1 Julius Center for Health Sciences and Primary Care, University Medical Center Utrecht and Utrecht University, Utrecht, The Netherlands; 2 Department of Cardiology, Maastricht University Medical Center, Maastricht, The Netherlands; 3 Department of Cardiology, Zuyderland Medical Center, Heerlen, The Netherlands; 4 Isala Hospital, Zwolle, The Netherlands; 5 Center for Integrated Care, Zwolle, The Netherlands; 6 Care Group Medrie, Zwolle, The Netherlands; 7 General Practice Assendorp, Zwolle, The Netherlands

**Keywords:** cardiovascular diseases, prevention, general practice, delivery of health care, integrated

## Abstract

**Background:**

Cardiovascular diseases (CVDs) are the leading cause of death worldwide. Despite the impact of CVDs, risk factors are often insufficiently controlled in patients at high risk. Recently, integrated multidisciplinary cardiovascular risk management (CVRM) programmes have been introduced in primary care.

**Aim:**

To investigate the effects of a CVRM programme on systolic blood pressure (SBP) and low-density lipoprotein (LDL)-cholesterol.

**Design & setting:**

A prospective observational study was undertaken in patients at high cardiovascular (CV) risk who were aged 40–80 years. Integrated CVRM care was compared with usual care in general practice in the Netherlands.

**Method:**

Intervention and usual care patients were matched at baseline on age, sex, and presence of CVD. During 1 year of follow-up, patients received integrated or usual CVRM care in general practice. Primary outcomes were SBP and LDL-cholesterol. Secondary outcomes included calculated 10-year CV risk, body mass index (BMI), lifestyle (smoking, physical activity, and dietary habits), medication use, patient satisfaction, healthcare consumption, morbidity, comorbidity, and mortality. Mixed-model analyses were used to assess the outcomes.

**Results:**

Totals of 372 and 317 patients were included in the intervention and usual care group, respectively. Mean age at baseline was 65.1 years and 66.2 years, respectively, and 42% were female in both groups. After 1 year, no differences were observed in: SBP (137.2 mmHg versus 139.0 mmHg in the intervention and usual care group, respectively); LDL-cholesterol (2.6 mmol/l in both groups); or in any of the secondary outcomes.

**Conclusion:**

Integrated CVRM care in general practice did not lead to a lower SBP or LDL-cholesterol in patients at high CV risk. Further research is needed to improve CVRM.

## How this fits in

In some European countries, integrated and multidisciplinary CVRM programmes have been introduced in primary care in recent years. Studies on the effectiveness of CVRM programmes are scarce and the available evidence is inconsistent. In the present study, 1 year of integrated primary care for CVRM following usual care did not lead to better outcomes for SBP (137.2 mmHg versus 139.0 mmHg) and LDL-cholesterol (2.6 mmol/l in both groups), or any of the secondary outcomes of the study, compared with usual care. This study adds relevant insight into effectiveness of integrated CVRM in a real-world environment and guides clinicians to look for improvements in the quality of CVRM programmes.

## Introduction

CVDs remain the leading cause of mortality worldwide.^1,2^ The European Society of Cardiology (ESC) recommends preventive, multidisciplinary programmes for CVRM, which should also be delivered in primary care.^3^ However, survey studies have shown that CVRM in primary care is suboptimally implemented, as control rates of CV risk factors are disappointing.^4–6^

In some European countries, integrated and multidisciplinary CVRM programmes have been introduced in primary care in recent years. Core elements of these programmes include: systematic selection; invitation; CV risk assessment; shared decision making in treatment and follow-up of eligible patients; stimulation of self-management; registration of patient data in clinical information systems; and yearly feedback to GPs on delivered CVRM care.^7^ So far, studies on the effectiveness of CVRM programmes are scarce and the available evidence is inconsistent.^8–10^

Some studies showed a trend towards improved lifestyle, but did not show an effect on CV risk factors and CV outcomes.^11–13^ However, the studies were heterogeneous in design, target population, and interventions tested, and adequate comparison with usual care was often lacking.

The present ZWOT-CASE study (ZWOlle inTegrated care for CArdiovaScular risk managEment study) reports the effects of the implementation of an integrated CVRM care programme on SBP and LDL-cholesterol in general practice compared with usual care.

## Method

### Design

A prospective observational study took place, comparing integrated care for CVRM with usual care during 1 year of follow-up. The details of the study design have been described elsewhere.^14^

### Setting

The study was performed in the Zwolle region in the Netherlands. It included 56 general practices, affiliated to a care group ‘Medrie’. All practices delivered usual care before the implementation of integrated care for CVRM. From January 2016, 37 general practices implemented integrated CVRM care and 19 general practices continued usual care. All practices were invited to participate in the study; 17 intervention and nine usual care practices participated.

### Patients

The aim was to include a total of 370 patients in each group consisting of, respectively: 1) 185 patients with CVD; and 2) 185 patients with a high (>10%) 10-year risk of CVD morbidity and mortality, based on the Dutch guideline for CVRM and a modifiable risk factor (SBP >140 mmHg, LDL-cholesterol >2.5 mmol/L, smoking, or BMI >30 kg/m^2^).^15^ It was ensured that 50% were aged <65 years and 50% ≥65 years. Inclusion and exclusion criteria are shown in Box 1.

Box 1 **Inclusion and exclusion criteria**

Inclusion criteria for patients with cardiovascular disease (CVD):

- patients with a history of atherosclerotic CVD, including angina pectoris, myocardial infarction, chronic ischaemic heart disease, coronary sclerosis, transient ischaemic attack (TIA), cerebral infarction, intermittent claudication, or aneurysm of the abdominal aorta;
- the patient is primarily managed by the GP; and
- aged 40–80 years.

Inclusion criteria for patients at high cardiovascular (CV) risk:

- no previous CVD;
- use of antihypertensive or lipid-lowering drugs; or
- a 10-year CV risk >10%, based on the Dutch guideline for CVRM, and: 1) either one strong CV-risk enhancing factor or two mild CV-risk enhancing factors (based on family history of CVD, physical activity, BMI, and renal function); or 2) >1 CV risk factor (current smoker, SBP >140 mmHg, LDL >2.5 mmol/l, total cholesterol [TC]/high-density lipoprotein [HDL]-ratio >8, chronic renal impairment [age <65 years: estimated glomerular filtration rate {eGFR} <60 ml/minute/1.73 m^2^; aged ≥65 years: eGFR <45 ml/minute/1.73 m^2^, and/or {micro}albuminuria]); or
- a 10-year CV risk of >20% and >1 CV risk factor, as mentioned above;
- at least one modifiable risk factor;
- the patient is primarily managed by the GP; and
- aged 40–80 years.

Exclusion criteria for all patients:

- diabetes mellitus (DM), as these patients receive CVRM in a DM programme;
- limited life expectancy;
- cognitive impairment;
- no Dutch language proficiency;
- staying abroad >3 months; and
- patient receives CVRM in the hospital or outpatient clinic from a medical specialist.

### Intervention

Implementation of the integrated CVRM programme was coordinated by the care group ‘Medrie’, in accordance with the regional hospital and the region's largest healthcare insurance company. It was based on the Dutch guideline for CVRM and the practical manual for CVRM provided by the Dutch Society of General Practitioners.^15,16^ GPs screened their practice population for eligible patients and invited them for an intake consultation for the integrated CVRM programme, which was mostly done by practice nurses (PNs) under supervision of GPs.^17^ During this consultation the researchers identified patients for the study. To prevent a Hawthorne effect, GPs and patients were not informed about the identification. Patients received the integrated CVRM programme as previously described.^14^ In short, before the intake, a blood sample was taken to measure lipids, renal function (Modification of Diet in Renal Disease [MDRD]), and glucose. The intake consultation included: assessment of CV complaints, lifestyle (smoking habits, diet, alcohol, and physical activity), and prescribed medication; measurement of blood pressure and BMI; estimation of the 10-year CV risk according to the Dutch guideline for CVRM in patients without CVD;^15^ and defining individual treatment goals in shared decisions. Patients were monitored at least once a year for control of CV risk factors. If necessary, other disciplines were involved, including dietitians, physiotherapists, and medical specialists. All disciplines had access to the patient data in the multidisciplinary information system, facilitating care coordination across organisations and ensuring a consistent policy in individual patients. After 1 year the study patients were revealed to the GP and they received a letter from their GP to inform them about the study. After agreement to participate, written informed consent was obtained during the endpoint visit.

### Usual care

Practices in the usual care group continued usual care. Patients were consecutively matched with intervention patients at baseline, on the basis of age (5-year categories), sex, and presence of CVD. Similar to the intervention group, the patients and their GPs were not informed about the identification. After 1 year, the matched patient was invited for a CVRM consultation if the corresponding intervention patient agreed to participate. Written informed consent was obtained and endpoints were measured during this consultation.

### Outcomes

The primary outcomes were SBP and LDL-cholesterol. Secondary outcomes included: diastolic blood pressure; achievement of treatment goals (blood pressure <140/90 mmHg, LDL-cholesterol <2.5 and <1.8 mmol/l for all patients and those with CVD, respectively); smoking status; BMI; 10-year CV morbidity or mortality risk (according to SMART and the Dutch guideline for CVRM, respectively);^15,18^ healthy food habits (according to the Dutch guideline for CVRM and the Dutch Health Council guideline on healthy food);^15,19^ alcohol consumption; physical activity (squash questionnaire);^20^ medication use (antihypertensive drugs, lipid-lowering drugs, and anticoagulants); primary treating practitioner in CVRM (GP or medical specialist); total number of consultations in general practice; patient satisfaction regarding the provided care (Patient Reported Experience Measure [PREM]); quality of life (EQ-5D and SF-12); anxiety and depression (Hospital Anxiety and Depression Scale [HADS]); and newly developed (co)morbidity and mortality.

### Data collection

Before the endpoint visit, patients filled out paper questionnaires (including the squash questionnaire, EQ-5D, SF-12, PREM, HADS, and food habits) and blood samples were taken for measurement of lipids, renal function, glucose, and high-sensitivity C-reactive protein (hs-CRP) for patients with CVD (to calculate SMART risk). During the endpoint visit PNs assessed office blood pressure,^16^ BMI, smoking status, alcohol consumption, and primary treating practitioner. After the endpoint visit, electronic medical records were manually scrutinised to assess baseline data, medication use, healthcare consumption, (co)morbidity and mortality, and whether a patient received previous CVRM care, defined as at least yearly visiting the general practice for a CVRM consultation, including measurement of lipids, renal function, and blood pressure.

All data relating to patients were pseudonymised.

### Sample size

The sample size was based on a 5.0 mmHg (standard deviation [SD] 15.9) absolute reduction in SBP and a 0.3 mmol/l (SD 1.0) reduction in LDL-cholesterol in the intervention group compared with usual care after 1 year of follow-up, with an alpha of 0.05, a power of 80%, and an intra-cluster correlation coefficient of 0.05 for the general practice cluster level. This led to a need of 370 patients in both groups. Accounting for a response rate of 70% in the intervention group, it was planned to invite 587 intervention patients. Anticipating a 50% response rate in the usual care group, each intervention patient was matched to two usual care patients, resulting in 587×2 = 1174 patients in the usual care group.

### Statistical analyses

Generalised linear mixed-model analyses were used. For continuous, count, and dichotomous outcomes, a linear, poisson and logistic distribution were assumed, respectively. For skewed distributed continuous outcomes, analyses were conducted with a logarithmic transformed variable, if appropriate, and the reversed logarithm of the B values and confidence intervals were calculated resulting in a ratio (interpreted as a multiplication factor).

Crude mixed-model analyses were used with a random intercept to correct for clustering within practices and additionally corrected for *a priori* defined potential confounding baseline covariates (use of antihypertensive and lipid-lowering drugs and anticoagulants, comorbidity [chronic obstructive pulmonary disease {COPD}, heart failure, atrial fibrillation, and renal failure], and practice characteristics [number of PNs, GPs, and patients]).

The authors examined potential effect modification of differences in practice characteristics (practice organisation [solo, duo, or group]), availability of CVRM protocol and existence of other disease management programmes (COPD and DM), and CVRM usual care given before the intervention (yes or no), by adding them as interaction terms to the crude model. If an interaction term was statistically significant (*P*<0.05), stratified analyses were conducted.

Statistical analyses were conducted in R studio (version 3.5.1).

## Results

**Figure 1.**
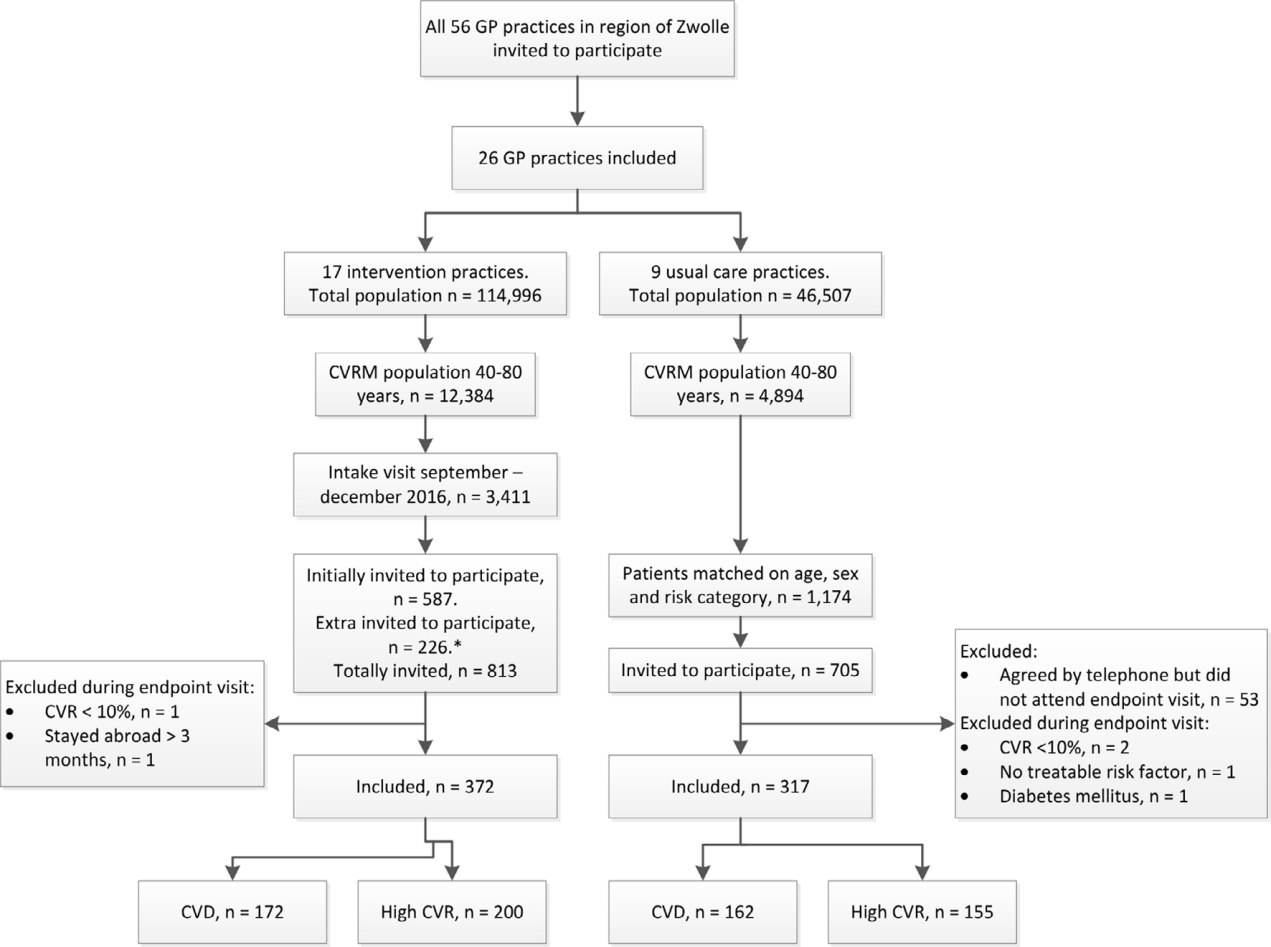
ZWOT-CASE study flow diagram. CVD = cardiovascular disease. CVR = cardiovascular risk. CVRM = cardiovascular risk management. ^*^As the response rate in the intervention group was lower than the expected 70%, the required sample size was not reached after 587 intervention patients were invited. Therefore, 226 extra patients were invited to the intervention group (total invited *n* = 813) and they were matched retrospectively to the usual care group.

In total, 689 patients were included: 372 intervention and 317 usual care patients (Figure 1). In the intervention and control group, 439 (62%) and 384 (54%) of the invited patients did not participate, respectively (50% and 45% were female, mean age was 63.5 years, and 39% had CVD in both groups).

Mean age in included patients was 65.1 years versus 66.2 years, respectively, and in both groups 42% were female (Table 1). At baseline, no differences were observed in CV risk factors, CVD, comorbidities, and medication use across the groups. Before the study, the proportion receiving CVRM care was higher in the intervention than in the usual care group (67% versus 51%, *P*<0.001).

**Table 1.** Baseline characteristics

Characteristics	Intervention group(*n* = 372)	Usual care group(*n* = 317)
Mean age, years (SD)	65.1 (8.3)	66.2 (7.5)
Age <65 years	175 (47)	132 (42)
Female	158 (42)	132 (42)
Western	358 (96)	295 (93)
**Cardiovascular risk factors**		
Hypertension^a^	280 (75)	234 (74)
Hypercholesterolemia^a^	91 (24)	91 (29)
Current smoker^b^	43 (12)	32 (10)
Chronic kidney disease^c^	40 (11)	51 (16)
Microalbuminuria^c^	15 (4)	10 (3)
Rheumatoid arthritis^a^	4 (1)	10 (3)
**Cardiovascular diseases^a,d^**	172 (46)	162 (51)
Myocardial infarction	41 (11)	48 (15)
Coronary sclerosis	46 (12)	44 (14)
Angina pectoris	44 (12)	39 (12)
Transient ischaemic attack	33 (9)	31 (10)
Cerebral infarction	35 (9)	17 (5)
Aneurysm aortae	8 (2)	11 (3)
Intermittent claudication	12 (3)	13 (4)
Atherosclerosis	4 (1)	4 (1)
**Comorbidities (including other CVD)^a^**		
COPD	9 (2)	14 (4)
Atrial fibrillation	23 (6)	16 (5)
Heart failure	1 (0.3)	3 (1)
**Medication use** **^b^**		
Antihypertensive agents	299 (80)	251 (79)
Statins/lipid-lowering agents	190 (51)	167 (53)
Anticoagulants	169 (45)	154 (49)
**Measurements^e^**		
Mean SBP, mmHg (SD)	136.7 (15.2)	—
Mean DBP, mmHg (SD)	80.3 (9.5)	—
Mean LDL-cholesterol, mmol/l (SD)	2.8 (0.9)	—
Mean BMI (SD)	27.7 (4.0)	—

^a^Based on International Classification of Primary Care (ICPC)-coded diagnoses. ^b^Based on medical records. ^c^Based on ICPC-coded diagnoses and/or laboratory measurements. Microalbuminuria: albumin-creatinine ratio >3 mg/mmol. Chronic kidney disease: ≥3 months impaired renal function (estimated glomerular filtration rate <60 ml/minute/1.73 m^2^) and/or microalbuminuria.^d^Cardiovascular diseases as inclusion criteria for integrated CVRM care and for the study. ^e^Baseline measurements of the control group at *t* = 0 are not presented, as there was no routine intake consultation.

BMI = body mass index. COPD = chronic obstructive pulmonary disease. CVD = cardiovascular diseases. DBP = diastolic blood pressure. LDL = low-density lipoprotein. SBP = systolic blood pressure. SD = standard deviation.

Absolute numbers (%) are presented unless stated otherwise.

In the intervention and usual care group, data were able to be collected on SBP in 96% and 94% of patients, and data on LDL-cholesterol in 93% and 98% of patients, respectively. Differences were not observed in both mean SBP and LDL-cholesterol between the intervention and usual care group at the endpoint (137.2 mmHg versus 139.0 mmHg, respectively, and 2.6 mmol/l in both groups) (Tables 2 and 3). None of the interaction terms for the primary outcomes were statistically significant (data not shown). Therefore, stratified analyses were not performed.

**Table 2.** Primary and secondary outcomes, descriptives

**Outcomes**	**Intervention group**(***n* = 372**)	**Usual care group**(***n* = 317**)
**Primary outcomes**	***N***		***N***	
Mean systolic blood pressure, mmHg (SD)	358^a^	137.2 (16.2)	298^b^	139.0 (16.8)
Mean LDL-cholesterol, mmol/l (SD)	347^c^	2.6 (0.8)	310^d^	2.6 (1.0)
**Secondary outcomes**				
Mean diastolic blood pressure, mmHg (SD)	358	80.3 (10.2)	298	80.6 (10.1)
Blood pressure <140/90 mmHg	358	214 (60)	298	175 (59)
LDL-cholesterol <2.5 mmol/l	347	178 (51)	310	168 (54)
LDL-cholesterol <1.8 mmol/l^e^	166	45 (27)	163	58 (36)
Smoking	363	31 (9)	311	30 (10)
Mean BMI (SD)	349	27.3 (5.2)	300	27.7 (4.8)
**10** **-year** **CVD morbidity or mortality risk^f^**				
All patients, median (IQR)	317	22.0 (11.7–36.4)	267	24.0 (13.7–38.0)
Patients with CVD, median (IQR)	159	26.2 (17.9–38.5)	144	27.8 (18.7–39.5)
Patients without CVD, median (IQR)	158	15.5 (5.4–31.9)	123	18.7 (8.4–34.3)
**Healthy food habits**				
Vegetables >150–200 grams a day	360	142 (39)	294	99 (34)
Fruits >200 grams a day	354	214 (60)	294	187 (64)
Red meat <300 grams a week	356	207 (58)	286	155 (54)
Fatty fish >1 a week	358	244 (68)	296	187 (63)
Unhealthy fat products <3 a week & healthy fat products >3 a week	352	121 (34)	289	75 (26)
Sweet & salty snacks <3 a week	357	196 (55)	295	157 (53)
Table salt <3 a week	360	335 (93)	294	265 (90)
Alcohol consumption, units a week, median (IQR)	311	3 (0–7)	292	2 (0–7)
Physically active^g^	303	230 (76)	250	178 (71)
**Medication use**				
**Patients with CVD**				
Antihypertensive drugs	174	137 (79)	160	121 (76)
Lipid-lowering drugs	174	139 (80)	160	127 (79)
Anticoagulants	174	160 (92)	160	146 (91)
**Patients without CVD**				
Antihypertensive drugs	187	167 (89)	149	126 (85)
Lipid-lowering drugs	188	52 (28)	149	46 (31)
GP as primary treating practitioner^h^	368	366 (99)	314	307 (98)
Consultations in general practice, median (IQR)^i^	361	6 (3–10)	311	6 (3–10)
Patient satisfaction (PREM) (1–5^j^), mean (SD)	359	3.6 (0.7)	283	3.5 (0.8)
Recommendation score (0–10^j^), mean (SD)	352	8.3 (1.3)	275	8.2 (1.3)
EQ-5D-5L index score (–0.45–1^j^), mean (SD)	353	0.9 (0.1)	290	0.8 (0.1)
SF-12 Mental component (7.9–72.0^j^), mean (SD)	353	53.9 (7.5)	290	52.3 (9.3)
SF-12 Physical component (5.2–64.7^j^), mean (SD)	353	48.1 (9.2)	290	46.7 (10.0)
HADS Anxiety (0–7^j^), mean (SD)	342	4.1 (3.3)	286	4.5 (3.7)
HADS Depression (0–7^j^), mean (SD)	347	3.2 (3.0)	283	3.9 (3.3)
Newly developed CVD^k^	364	10 (3)	311	10 (3)
Newly developed comorbidity^l^	363	13 (4)	311	12 (4)
Mortality	372	5 (1)	318	3 (1)

^a^Reasons for missing data: died before endpoint (*n* = 5), not measured (*n* = 7), data not available owing to change of GP (*n* = 2). ^b^Reasons for missing data: died before endpoint (*n* = 3), not measured (*n* = 16). ^c^Reasons for missing data: died before endpoint (*n* = 5), not measured (*n* = 16), data not available owing to change of GP (*n* = 4). ^d^Reasons for missing data: died before endpoint (*n* = 3), not measured (*n* = 3), data not available owing to change of GP (*n* = 1). ^e^For patients with CVD, *n* = 175 in intervention group and *n* = 164 in usual care group. ^f^For patient with known CVD the SMART-function was used to calculate the risk; for patients without CVD the risk was based on the risk chart in the Dutch guideline for CVRM (based on the SCORE risk function).^15^ ^g^ >5 days a week moderate intense physical activity >30 minutes a day. ^h^Primary treating practitioner could be the GP or a medical specialist. ^i^Including all visits and telephone calls with the general practice for all reasons. ^j^Minimum and maximum possible values. ^k^Including cardiovascular diseases as inclusion criteria for integrated CVRM care and for the study. ^l^Including diabetes mellitus, chronic obstructive pulmonary disease, heart failure, atrial fibrillation, and chronic renal impairment.

BMI = body mass index. CVD = cardiovascular diseases. EQ-5D-5L = five-level EuroQoL-5 Dimensions. HADS = Hospital Anxiety and Depression Scale. IQR = interquartile range. LDL = low-density lipoprotein. PREM = Patient Reported Experience Measure. SD = standard deviation. SF-12 = Short Form–12 Health Survey.

Absolute numbers (%) are presented unless stated otherwise.

**Table 3.** Effect of integrated CVRM care on the primary and secondary outcomes compared with usual care, using generalised mixed-model analyses

		**Crude model^a^**		**Adjusted model^b^**
**Outcomes**	***n***	**Beta^c^**	**95%** **CI**	***P*** **value**	***n***	**Beta^c^**	**95%** **CI**	***P*** **value**
**Primary outcomes**								
Systolic blood pressure	656	–1.75	–5.78 to 2.29	0.38	647	–1.78	–6.09 to 2.53	0.40
LDL-cholesterol	657	0.05	–0.13 to 0.23	0.58	653	0.01	–0.15 to 0.18	0.86
**Secondary outcomes, continuous**								
Diastolic blood pressure	656	0.04	–3.05 to 3.13	0.97	647	–0.37	–3.78 to 3.04	0.82
BMI	649	–0.27	–1.28 to 0.74	0.59	641	0.09	–0.83 to 1.02	0.84
EQ-5D-5L index score	643	0.01	–0.02 to 0.04	0.46	633	0.01	–0.02 to 0.03	0.64
SF-12 Mental component	643	1.61	0.21 to 3.02	0.03^d^	633	1.39	–0.17 to 2.95	0.08
SF-12 Physical component	643	1.45	–0.38 to 3.28	0.12	633	1.01	–0.74 to 2.76	0.25
Patient satisfaction (PREM)	642	0.13	–0.03 to 0.29	0.11	631	0.14	–0.03 to 0.32	0.10
Recommendation score	627	0.13	–0.10 to 0.36	0.24	616	0.11	–0.13 to 0.36	0.35
HADS Anxiety	628	–0.39	–1.05 to 0.27	0.23	618	–0.35	–1.06 to 0.37	0.32
HADS Depression	630	–0.61	–1.27 to 0.06	0.07	620	–0.45	–1.19 to 4.41	0.22
	***n***	**Ratio** **^e^**	**95%** **CI**	***P*** **value**	***n***	**Ratio** **^e^**	**95%** **CI**	***P*** **value**
**Secondary outcomes, log transformed**								
10-year CV risk								
All patients	584	0.87	0.75 to 1.02	0.08	583	0.90	0.76 to 1.06	0.21
Patients with CVD	303	0.98	0.86 to 1.12	0.76	303	1.04	0.90 to 1.20	0.59
Patients without CVD	281	0.81	0.62 to 1.06	0.11	280	0.80	0.60 to 1.08	0.15
**Secondary outcomes, dichotomous**								
Blood pressure <140/90 mmHg	656	0.96	0.54 to 1.83	0.99	647	0.97	0.52 to 1.83	0.93
LDL-cholesterol <2.5 mmol/l	657	0.89	0.64 to 1.23	0.48	653	1.13	0.74 to 1.72	0.57
LDL-cholesterol <1.8 mmol/l	329	0.64	0.37 to 1.13	0.12	326	0.70	0.39 to 1.26	0.24
Smoking	674	0.87	0.52 to 1.48	0.62	671	1.00	0.54 to 1.85	0.99
**Healthy food habits^f^**								
Vegetables	654	1.28	0.93 to 1.77	0.13	643	1.26	0.87 to 1.83	0.21
Fruits	648	0.88	0.64 to 1.20	0.41	637	0.94	0.65 to 1.35	0.72
Red meat	642	1.17	0.86 to 1.61	0.32	632	1.21	0.85 to 1.74	0.29
Fatty fish	654	1.30	0.89 to 1.91	0.18	643	1.24	0.83 to 1.85	0.30
Fatty products	641	1.49	1.06 to 2.11	0.02^d^	631	1.41	0.95 to 2.08	0.09
Snacks	652	1.07	0.79 to 1.46	0.67	641	1.22	0.85 to 1.73	0.28
Table salt	654	1.47	0.84 to 2.58	0.18	643	1.68	0.84 to 3.35	0.14
Physical activity	553	1.29	0.83 to 1.99	0.25	543	1.31	0.84 to 2.06	0.24
**Medication use**								
**Patients with CVD**								
Antihypertensive drugs	334	1.19	0.72 to 1.99	0.50	334	5.09	0.56 to 46.0	0.15
Lipid-lowering drugs	334	1.03	0.61 to 1.76	0.91	334	0.97	0.26 to 3.57	0.96
Anticoagulants	334	1.10	0.51 to 2.38	0.82	334	1.52	0.23 to 9.90	0.66
**Patients without CVD**								
Antihypertensive drugs	336	1.52	0.80 to 2.90	0.20	336	1.23	0.12 to 12.2	0.86
Lipid-lowering drugs	337	0.86	0.53 to 1.37	0.52	337	0.71	0.18 to 2.79	0.62
GP as primary treating practitioner	682	3.93	0.74 to 21.0	0.11	671	10.50	0.80 to 138.3	0.07
Newly developed CVD	675	0.85	0.35 to 2.07	0.72	671	0.99	0.37 to 2.64	0.99
Newly developed comorbidity	674	0.91	0.34 to 2.41	0.85	671	1.11	0.43 to 2.91	0.83
Mortality	689	1.48	0.32 to 6.89	0.62	672	0.37	0.00 to 38.8	0.68
**Secondary outcomes, count**								
Alcohol consumption	601	0.88	0.65 to 1.19	0.39	594	0.81	0.60 to 1.09	0.17
Consultations in general practice	672	1.05	0.89 to 1.25	0.54	670	1.04	0.89 to 1.21	0.65

^a^Corrected for clustering within practices. ^b^Corrected for clustering within practices and predefined confounders. ^c^Difference between intervention and usual care group. ^d^Statistically significant. ^e^Ratio, should be interpreted as a multiplication factor. For example, a ratio of 1.05 should be interpreted as a 5% higher outcome score in the intervention group compared with the usual care group. ^f^Healthy food habits: vegetables >150–200 grams a day; fruits >200 grams a day; red meat <300 grams a week; fatty fish >1 a week; unhealthy fatty products <3 a week and healthy fatty products >3 a week; sweet and salty snacks <3 a week; table salt <3 a week.

BMI = body mass index. CV = cardiovascular. CVD = cardiovascular disease. EQ-5D = five-level EuroQoL-5 Dimensions. HADS = Hospital Anxiety and Depression Scale. LDL = low-density lipoprotein. PREM = Patient Reported Experience Measure. SF-12 = Short Form–12 Health Survey.

Treatment goals for blood pressure and LDL-cholesterol were achieved in slightly more than half of the patients in both groups (Table 2); 60% versus 59% reached a blood pressure target of <140/90 mmHg and 51% versus 54% achieved target of LDL-cholesterol <2.5 mmol/l. In patients with CVD, 27% versus 36% reached a LDL-cholesterol <1.8 mmol/l. Smoking rates were 9% versus 10%, respectively. BMI, CV risk, physical activity, alcohol consumption, and food habits did not differ between both groups. Approximately one-third of participants in both groups achieved healthy food habits regarding vegetables and fats, and 53%–68% reported a healthy dietary pattern concerning intake of fruit, red meat, fatty fish, and snacks. No differences were observed between the groups in terms of medication use, number of consultations during follow-up (median 6), satisfaction with the delivered care (median 3.6 and 3.5, respectively, on a scale of 1–5), and recommendation scores to their GP (median 8.3 and 8.2, respectively, on a scale of 0–10). Similar results were observed for quality of life and anxiety and depression scores.

## Discussion

### Summary

In this observational study, 1 year of integrated primary care for CVRM following usual care did not lead to better outcomes for SBP and LDL-cholesterol, or any of the secondary outcomes of the study, compared with usual care.

### Strengths and limitations

Strengths of the ZWOT-CASE study are its prospective design, the real-world setting, the matched groups from the same environment, the reliably measurable outcomes, and reasoned statistical methods. However, the lack of random allocation to the two study arms may have led to confounding bias. Ample measures were taken (notably matching of patients and multivariable analyses) to prevent and correct for confounding, and baseline characteristics were comparable between both groups. Also, it was found that care given before implementation of integrated CVRM care did not affect the effect of the intervention. Although residual confounding is possible, it is believed that the observational design of the study is of large value, as randomisation of regionally implemented complex interventions is hardly possible.

All general practices in this study were from the region of Zwolle and affiliated to the care group ‘Medrie’. Therefore, usual care may have changed in the direction of the intervention and consequently the effect of the intervention may have been underestimated. However, this setting reflects real practice as integrated CVRM care is always implemented regionally in the Netherlands.

Another limitation is the lower statistical power than calculated *a priori* owing to the 14% lower participation rate in the usual care group. However, a post-hoc power analysis showed that the authors still would have been able to find a difference of 3.65 mmHg in SBP, which they consider as still clinically relevant.

In both groups, the response rates were lower than expected. It is assumed that reasons for (non)-participation were similar in both groups, but it cannot be ruled out that this may have led to some bias.

Finally, there were some missing outcome data in both groups. Since missing data were not extensively present in the primary outcomes and differences were not observed in missing endpoints between both groups, it is expected that imputation would not change the results.

### Comparison with existing literature

The results are in line with previous studies, showing disappointing findings.^9^ One Dutch study on the effect of disease management programmes (DMPs) for CVRM in general practice, showed a trend towards improved lifestyle (increased physical activity and reduced smoking) after 2 years.^13^ However, this study included a heterogeneous population (some DMPs targeted only patients with CVD, some included patients at high-risk without CVD as well) and comparison with usual care and assessment of clinical outcomes (SBP and lipids) was lacking.

A cluster randomised controlled trial (RCT) compared a tailored implementation of CVRM in general practice to usual care and found a significant improvement in physical activity, but not in other outcomes (SBP, LDL-cholesterol, smoking status, BMI, and diet) after 6 months.^12^ However, this intervention is not easily comparable to the present study as it focused on motivational interviewing, online education for PNs, and e-health options for patients.

The follow‐up time of the current study was shorter than the follow-up in a Dutch cluster RCT (1 year versus 5.4 years). In that study, a CVRM programme in primary care significantly reduced SBP with 2.39 mmHg in older adults (aged 70–78 years) without CVD. However, this reduction was largely obtained in the first year of follow-up.^21^ This suggests that an effect after 1 year would have been able to have been observed. However, it is known that it takes time to implement a new programme and to improve health care as practices have to adapt to new standards of quality and reorganise their practice.^22^ Therefore, it can’t be ruled out that a longer follow-up time would have resulted in better outcomes.

Comparison with other studies is difficult, given the heterogeneity in study design, interventions tested, outcomes measured, and target populations. Overall, most studies point towards no robust effect on CV risk factors or outcomes.^9,23^

Several reasons could explain the lack of effectiveness. First, the intervention itself could be ineffective owing to insufficient intensity, lack of personalised health promotion, or multidisciplinary collaboration. Possibly, PNs are insufficiently prepared, as their workload increases and patients become more complex.^24^

Besides, intensifying medication according to the guidelines if needed may have failed.^25^ Although GPs received yearly feedback on the state of CV risk factors of their CVRM population in the intervention group, not achieving treatment goals had no consequences. A reward system might enhance risk factor control and a continuous feedback system could improve CVRM in daily practice.^25^ Further, patient-related factors, such as inadequate risk and lifestyle perception, non-adherence to lifestyle advice, and medication, could have played a role. More insight into the patient perspective of CVRM care could lead to better communication about CV risk, more patient empowerment, and, possibly, better adherence to the advised therapy.^26^ Moreover, the efforts of primary care need support from government and society regarding lifestyle improvement.^9^

A second reason could be that usual care was already of high quality, diminishing the contrast between the intervention and usual care. As more than half of the patients in both groups received usual CVRM care previously to the present study, the largest reduction in SBP and LDL-cholesterol may already have been gained, which would appear to leave little room for further improvement. However, there is room for improvement, as <60% reached blood pressure and LDL-targets.

### Implications for research and practice

Despite the lack of effect, the potential of CVRM programmes to reduce CV risk should not be depreciated, but potentials to improve their quality should be focused on. To help reshape CVRM in primary care, a process evaluation is needed to provide a deeper understanding of the lack of effectiveness of the intervention in the present study. For GPs participating in a programme for CVRM, it is recommended to critically evaluate the process of care in their daily practice, and to organise direct and adequate feedback regarding adherence to CVRM guidelines, if possible supported by information and communication technology.

Furthermore, the effect of CVRM programmes in countries with lower quality of CVRM in usual primary care should be evaluated, as they may be more effective there.^27^ Also, out-of-the-box strategies to organise CVRM care should be considered, for example, other settings than general practice or a more multidisciplinary approach. Finally, modernisation of prevention programmes, for example by a more continuous telemetric risk factor control, may be promising.^8^

## Trial registration number

The ZWOlle Transmural Integrated Care for CArdiovaScular Risk Management Study; ClinicalTrials.gov; Identifier: NCT03428061; date of registration: 09-02-2018.
